# The Metabolic Reprogramming of *Frem2* Mutant Mice Embryos in Cryptophthalmos Development

**DOI:** 10.3389/fcell.2020.625492

**Published:** 2021-01-08

**Authors:** Xiayin Zhang, Ruixin Wang, Ting Wang, Xulin Zhang, Meimei Dongye, Dongni Wang, Jinghui Wang, Wangting Li, Xiaohang Wu, Duoru Lin, Haotian Lin

**Affiliations:** ^1^State Key Laboratory of Ophthalmology, Zhongshan Ophthalmic Center, Sun Yat-sen University, Guangzhou, China; ^2^Center for Precision Medicine, Sun Yat-sen University, Guangzhou, China

**Keywords:** metabonomics, *Frem2* mutation, cryptophthalmos, development of eyelids, transcriptomics

## Abstract

**Background:**

Cryptophthalmos is characterized by congenital ocular dysplasia with eyelid malformation. The pathogenicity of mutations in genes encoding components of the FRAS1/FREM protein complex is well established, but the underlying pathomechanisms of this disease are still unclear. In the previous study, we generated mice carrying *Frem2*^*R725X/R2156W*^ compound heterozygous mutations using CRISPR/Cas9 and showed that these mice recapitulated the human cryptophthalmos phenotype.

**Methods:**

In this study, we tracked changes in the metabolic profile of embryos and expression of metabolism-related genes in *Frem2* mutant mice on E13.5 compared with wild-type mice. RNA sequencing (RNA-seq) was utilized to decipher the differentiated expression of genes associated with metabolism. Untargeted metabolomics and targeted metabolomics analyses were performed to detect and verify the shifts in the composition of the embryonic metabolome.

**Results:**

Differentially expressed genes participating in amino acid metabolism and energy metabolism were observed by RNA-seq. Transcriptomic analysis suggests that 821 (39.89%) up-regulated genes and 320 (32.99%) down-regulated genes were involved in the metabolic process in the enriched GO terms. A total of 92 significantly different metabolites were identified including creatine, guanosine 5′-monophosphate, cytosine, cytidine 5′-monophosphate, adenine, and L-serine. Interestingly, major shifts related to ATP binding cassette transporters (ABC transporters) and the biosynthesis of amino acids in the composition of the embryonic metabolome were observed by KEGG metabolic analysis, indicating that these pathways could also be involved in the pathogenesis of cryptophthalmos.

**Conclusion:**

We demonstrate that *Frem2* mutant fetal mice have increased susceptibility to the disruption of eye morphogenesis in association with distinct transcriptomic and metabolomic signatures. Our findings suggest that the metabolomic signature established before birth may play a role in mediating cryptophthalmos in *Frem2* mutant mice, which may have important implications for the pathogenesis of cryptophthalmos.

## Introduction

Cryptophthalmos (MIM: 123570) is a rare congenital ocular dysplasia accompanied by fusion of the eyelids (François, 1969). Mutations in the *FREM2* gene result in reduced epithelial-mesenchymal coupling and transient embryonic epidermal blistering, leading to the development of cryptophthalmos (Jadeja et al., 2005; Haelst et al., 2010). However, the underlying pathomechanisms of this disease are still not known.

Metabolomics is considered as a sensitive approach for the development of novel targeted therapeutics because of its direct elucidation of pathophysiological mechanisms and its mediation as signals that directly or indirectly trigger adaptive responses (Piazza et al., 2018). Nutritional states, stress, and ecological conditions can all influence the intracellular levels of metabolites, and the development of the eye has been proven to be associated with intermediates of multiple metabolites by accumulating evidence (Robciuc et al., 2013; Cherif et al., 2018; Paramita et al., 2018). It is unclear whether the absence of metabolites drives epithelial–mesenchymal coupling in either mice or humans and whether the metabolic disorder is a consequence of cryptophthalmos.

Our objective, therefore, was to define the contribution of the metabolic disorder in driving cryptophthalmos as a predisposing factor. In our previous study, we established a mouse model that recapitulates the human complete cryptophthalmos phenotype characterized by abnormal eyelids, microphthalmia, and severe ocular anterior segment developmental defects (Zhang et al., 2019). Here, we used untargeted metabolomics analysis in conjunction with RNA-seq, to examine the contribution of metabolites and metabolism-related genes in modulating cryptophthalmos susceptibility in *Frem2* mutant fetal mice. Real-time quantitative PCR and targeted metabolomics analysis were performed to verify the shifts in the composition of the embryonic metabolome. We suggest that the metabolites established before birth may have an impact on cryptophthalmos susceptibility in *Frem2* mutant mice.

## Materials and Methods

### Animals

All animals used in this study were *Mus musculus*, C57BL/6J mice. All mouse procedures were approved by the Institutional Animal Care and Use Committee of Zhongshan Ophthalmic Center, Sun Yat-sen University. All experiments were carried out in accordance with the ARVO Statement for the Use and Care of Animals in Ophthalmic and Vision Research. The mice were maintained on a 12-h: 12-h light-dark cycle with unlimited access to food and water. All animals used for experiments were killed humanely and quickly by cervical dislocation (adult mice) or decapitation (embryos).

The generation of mice carrying the c.2173C > T (R725X) or c.6466A > T (R2156W) point mutation in the murine *Frem2* gene was designed as previously reported using CRISPR/Cas9 technology (Zhang et al., 2019). According to our previous study, the phenotypes exhibited by the *Frem2*^*R725X/R2156W*^ adult mice were bilateral or unilateral cryptophthalmos. Furthermore, 8/11 mice exhibited a single kidney and 4/11 mice exhibited syndactyly (Zhang et al., 2019).

### Histological Analysis

The embryos from *Frem2* mutant and wild-type mice were fixed with 4% paraformaldehyde and subsequently embedded in paraffin for at least 24 h. Tissues were sectioned in a vertical pupillary optic nerve plane and stained with H&E.

### RNA-Seq Analysis and Real-Time Quantitative PCR Analysis

We compared embryonic transcriptome changes between three *Frem2* mutant mice and one wild-type littermates. Total RNA was extracted with TRIzol reagent (Invitrogen). The RNA-seq libraries were generated using Illumina TruSeq RNA Sample Preparation Kits. RNA-seq was performed using an Illumina HiSeq 2000 platform as previously reported (Robciuc et al., 2013). Genes with q ≤ 0.05 and | log2_ratio| ≥ 1 were identified as differentially expressed genes. GO enrichment analyses of the differentially expressed genes were performed using DAVID.^1^ GO terms with a Bonferroni corrected *P* value of < 0.05 were considered significantly enriched functional annotations.

Real-time quantitative PCR was performed in three *Frem2* mutant mice embryos and three wild-type embryos on a 7900HT Real-time PCR system (Applied Biosystems) using real-time primers and TaqMan probes from Applied Biosystems. The first strand was reverse-transcribed using the Omniscript Reverse Transcription Kit (Qiagen) and random primers. The fold changes in gene expression were calculated using the ΔΔCt (cycle threshold) values. Expression was normalized to *Actin*. All primers used are listed in Supplementary Table S1.

### Untargeted Metabolomics Analysis

Untargeted high-throughput metabolomic profiling was conducted in six embryos of *Frem2* mutant mice and compared with that in six wild-type embryos. Frozen embryos of *Frem2*^*R725X/R2156W*^ and wild-type mice were thawed and analyzed using an UHPLC (1290 Infinity LC, Agilent Technologies) coupled with an AB SCIEX Triple TOF 6600 System (AB SCIEX, Framingham, MA, United States). In parallel to the preparation of the test samples (in a group of six), pooled quality control samples were prepared by mixing equal amounts (30 μL) of each sample. The quality control samples were utilized to monitor the LC-MS response in real time.

All samples were analyzed using a 2.1 mm × 100 mm ACQUIY UPLC BEH 1.7 μm column (Waters, Ireland). In both ESI positive and negative modes, the mobile phase contained the following: A, 25 mM ammonium acetate, and 25 mM ammonium hydroxide in water; B, acetonitrile (Merck, 1,499,230–935). The gradient elution program was 95% B for 1 min, linearly reduced to 65% in 13 min, reduced to 40% in 2 min, kept for 2 min, and then increased to 85% in 0.1 min, with a 5 min re-equilibration.

The ESI source conditions were set as follows: ion source gas1, 60; ion source gas2, 60; curtain gas, 30; source temperature, 600°C, ion spray voltage floating ± 5500 V, TOF MS scan *m/z* range, 60–1000 Da; and accumulation time, 0.20 s/spectra; production scan *m/z* range, 25–1000 Da; and accumulation time, 0.05 s/spectra for MS/MS acquisition. The production scan is acquired using information-dependent acquisition by high sensitivity mode with collision energy as 35 V with ±15 eV and declustering potential as ±60 V.

The raw MS data (wiff. scan files) were converted to mzXML files through ProteoWizard and processed by XCMS. The metabolites were identified by accuracy mass (<25 ppm) and MS/MS data that were matched with the lab database (Shanghai Applied Protein Technology Co., Ltd.). The variables having more than 50% of the valid values in at least one group were kept and handled for the multivariate statistical analysis by MetaboAnalyst^2^, as well as unidimensional statistical analysis by R software. The significantly different metabolites were determined based on the combination of a statistically significant threshold of VIP values obtained from supervised partial least squares-discriminate analysis and a two-tailed Student’s *t*-test. The VIP can measure the impact strength and interpretation capability of each metabolite pattern to distinguish samples in each group. Metabolites with VIP > 1.0 and *P* < 0.1 were considered significant. For metabolite annotation and pathway analysis, metabolites that showed differences between groups were analyzed by the KEGG^3^ database.

### Targeted Metabolomics Analysis

Targeted high-throughput metabolomic profiling was conducted in five embryos of *Frem2* mutant mice and compared with that in six wild-type mice. To precipitate proteins, the samples were incubated for 1 h at −20°C, followed by 15 min centrifugation at 13,000 r/min and 4°C. The resulting supernatant was removed and evaporated to dryness in a vacuum concentrator. The dry extracts were then reconstituted in 100 mL ACN: H2O (1:1, v/v), sonicated for 10 min, and centrifuged for 15 min at 13,000 r/min and 4°C to remove insoluble debris. The supernatants were transferred to HPLC vials for LC-MS analysis. The LC-MS analysis was performed using a UHPLC system (Agilent Technologies) coupled to a triple quadrupole mass spectrometer in the MRM mode. For the argininosuccinate and arginine tests alone, metabolites were monitored in positive mode only. For high-throughput metabolic profiling, metabolites were monitored in both ESI positive and ESI negative modes. A WATERS ACQUITY UPLC BEH Amino column (particle size, l.7 mm, 100 mm length × 2.1 mm ID) was used for argininosuccinate and arginine tests. Phenomenex Luna amino column (particle size, 3 mm; 100 mm length 3 2.1 mm ID) was used for metabolic profiling. The column temperature was kept at 25°C. The flow rate was 300 mL/min and the sample injection volume was 2 mL. Mobile phase A was 25 mM ammonium acetate and 25 mM ammonium hydroxide in 100% water, and B was 100% acetonitrile. The linear gradient for argininosuccinate and arginine tests was set as follows: 0–1 min: 85% B, 1–6 min: 85% B to 70% B, 6–10 min: 70% B to 0% B, 10–15 min: 0% B, 15–15.1 min: 0% B to 85% B, 15.1–20 min: 85% B. The linear gradient for metabolic profiling was set as follows: 0–1 min: 85% B, 1–14 min: 95% B to 65% B, 14–16 min: 65% B to 40% B, 16–18 min: 40% B, 18–18.1 min: 40% B to 95% B, 18.1–23 min: 95% B. ESI source conditions were set as follows: sheath gas temperature, 350°C; dry gas temperature, 350°C; sheath gas flow, 12 L/min for argininosuccinate and arginine tests, 11 L/min for metabolic profiling; dry gas flow, 16 L/min for argininosuccinate and arginine tests, 10 L/min for metabolic profiling; capillary voltage, 3000 V in positive mode for argininosuccinate and arginine tests, 4000 or −3500 V in positive or negative modes for metabolic profiling; nozzle voltage, 1000 V for argininosuccinate and arginine tests, 500 V for metabolic profiling; nebulizer pressure, 40 psi for argininosuccinate and arginine tests, 30 psi for metabolic profiling. For argininosuccinate and arginine tests, four MRM transitions representing the two metabolites were simultaneously monitored. The dwell time for each MRM transition is 50 ms, and the total cycle time is 535 ms. For metabolic profiling, the dwell time for each MRM transition is 3 ms, and the total cycle time is 1.263 s. Original MRM raw data were processed by MRMAnalyzer based on detection and area integration of peaks from individual target metabolites. Protein concentration was used for sample normalization.

To construct the metabolite MRM library, each metabolite standard (100 mg/mL) was first analyzed in ESI positive/negative mode via flow injection using the software MassHunter Optimizer (Agilent Technologies) to obtain the optimal MRM transition parameters. Then the retention time of each metabolite was determined by measuring the corresponding MRM transition individually on the column. The significantly different metabolites were determined based on the combination of a statistically significant threshold from fold change analysis (fold change > 1.5) and a two-tailed Student’s *t*-test (*P* < 0.05).

### Statistical Analysis

The principal component analysis was performed to examine intrinsic clusters of metabolomics data. Heatmaps were generated using a hierarchical clustering algorithm to visualize metabolite differences. All statistical analyses were performed using SPSS (version 19.0) and R (version 3.3.1). Significance levels of *P* values are graphically represented with: ^∗^ for *P* values between 0.05 and 0.01.

## Results

### Ocular Abnormalities in *Frem2* Mutant Embryos

**FIGURE 1:**
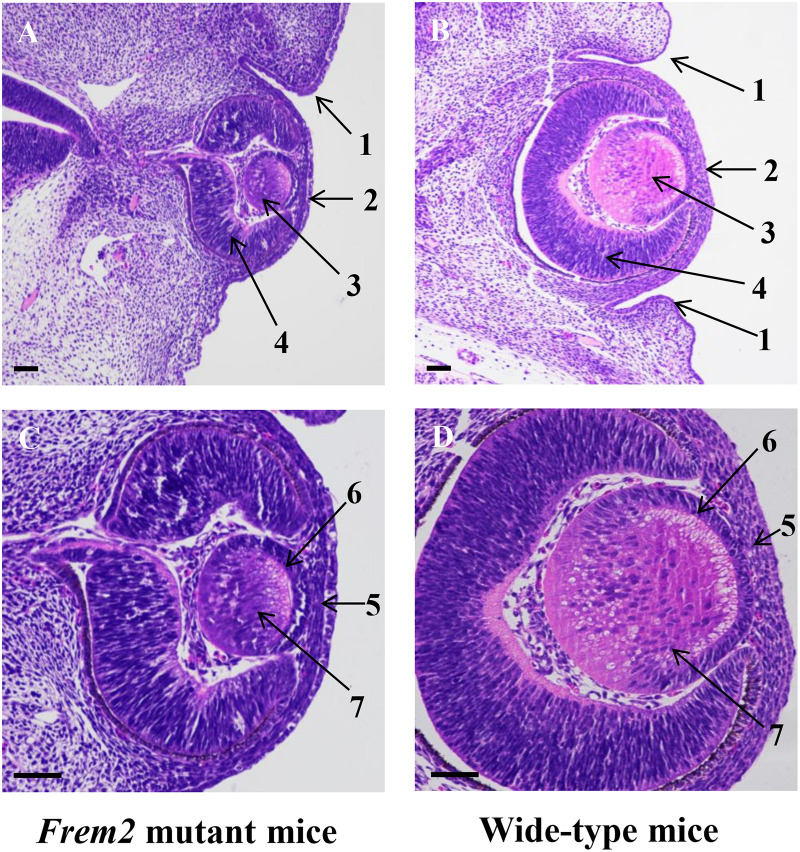
Abnormal ocular development in fetal *Frem2* mutant mice. Histology of eyes from E13.5 *Frem2*^*R725X/R2156W*^ mice **(A,C)** and wild-type mice **(B,D)**. **(A)** The lower eyelid fold cannot be defined in *Frem2* mutant mice, and the axial length and size of the lens are significantly reduced. **(C)** The corneal epithelium stratifies unclearly with the reduction in corneal epithelial thickness. The primary lens fibers are significantly shortened. Numbered arrows refer to the following: 1. eyelid; 2.cornea; 3. lens vesicle; 4. retina; 5. cornea epithelium; 6. lens epithelium; 7. primary lens fiber. Scale bars in all panels = 200 μm.

As formation of the eyelids in the mouse begins at approximately 13–14 days of gestation (Pei and Rhodin, 1970), we studied E13.5 mice. H&E staining confirmed microphthalmia and showed dysplastic eye structures in the *Frem2* mutant embryo (Figure 1). At the beginning of eyelid formation, the lower eyelid fold cannot be defined in the *Frem2* mutant embryo, whereas small grooves developing from the surface ectoderm to form the eyelid folds above and below the developing eye have been well formed in wild-type mice. The corneal epithelium of the *Frem2* mutant embryo stratifies unclearly with the reduction in corneal epithelial thickness. In addition, the dysplastic eyes exhibited a reduced axial length and reduced lens thickness compared to the wild-type mice.

### Identification of Differentially Expressed Genes Enriched in the Metabolic Process

To identify other genes that potentially contribute to the ocular phenotype, RNA-seq was performed with RNA from the whole embryos of E13.5 mice. RNA-seq results confirmed decreased expression of *Frem2* and genes contributing to extracellular matrix organization and cell-matrix adhesion, including *Ecm2*, *Col6a1*, *Col5a3*, *Colla1*, and *Lama5* in *Frem2* mutant mice (Zhang et al., 2019). In addition, we observed differentially expressed genes including *Stat5a*, *Cbs*, *Dpys*, *Adssl1*, and *Pm20d1* participating in amino acid metabolism, and *Ldhb*, *Hk1*, *Chchd10* participating in energy metabolism in fetal *Frem2*^*R725X/R2156W*^ mice.

**FIGURE 2:**
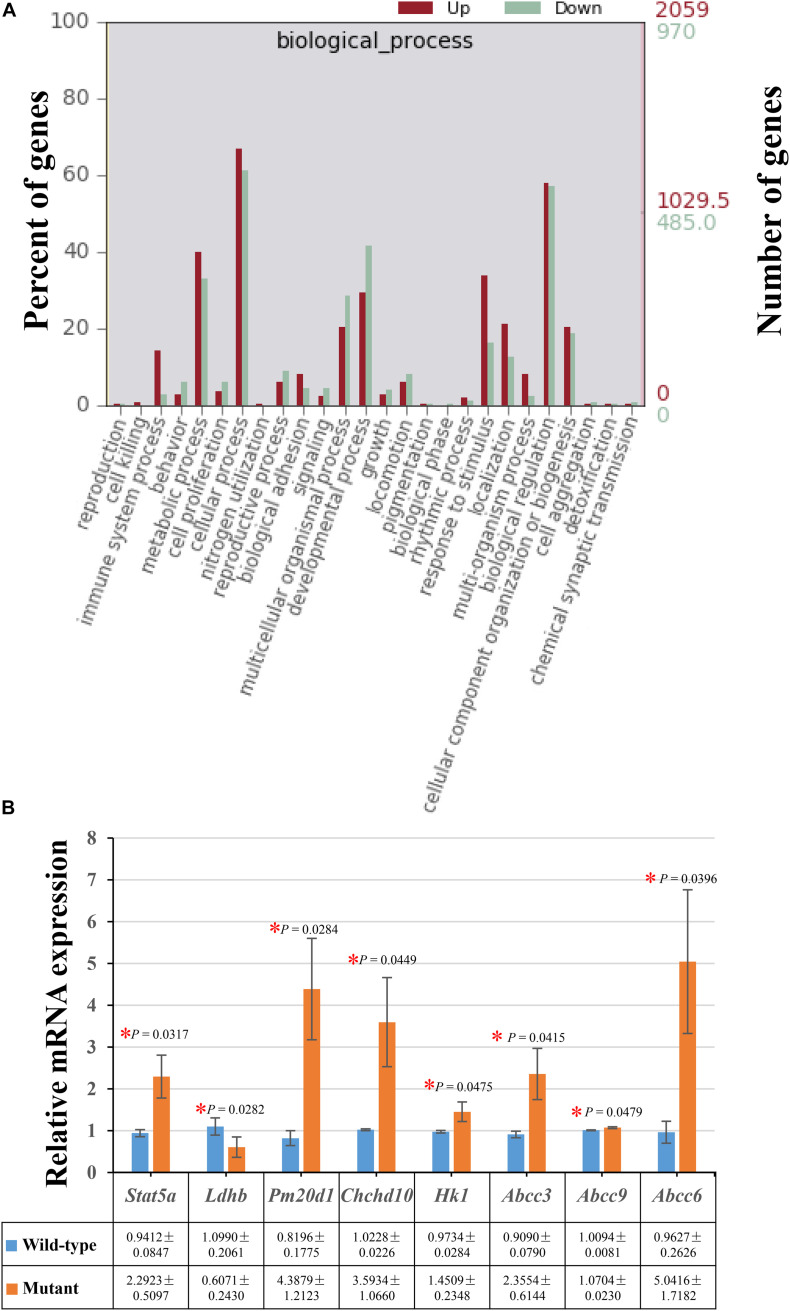
Differentially expressed genes enriched in the metabolic process. **(A)** A total of 821 (39.89%) up-regulated genes and 320 (32.99%) down-regulated genes involved in the metabolic process were identified in the enriched GO terms of biological processes. **(B)** The decreased expressions of *Ldhb*, and increased expression levels of *Stat5a*, *Pm20d1*, *Chchd10*, *Hk1*, *Abcc3*, *Abcc9*, and *Abcc6* were verified by performing real-time quantitative PCR in the whole embryos of *Frem2*^*R725X/R2156W*^ mice (*P < 0.05). Data are presented as the mean ± standard deviation (*n* = 3).

Moreover, there were 821 (39.89%) up-regulated genes and 320 (32.99%) down-regulated genes involved in the metabolic process in the enriched GO terms of biological processes (Figure 2A). The decreased expressions of *Ldhb* and increased expression levels of *Stat5a*, *Pm20d1*, *Chchd10*, and *Hk1* were verified by performing real-time quantitative PCR in the embryos of *Frem2*^*R725X/R2156W*^ mice (Figure 2B).

### *Frem2* Mutant Fetal Mice Have a Distinct Metabolomic Profile

**FIGURE 3:**
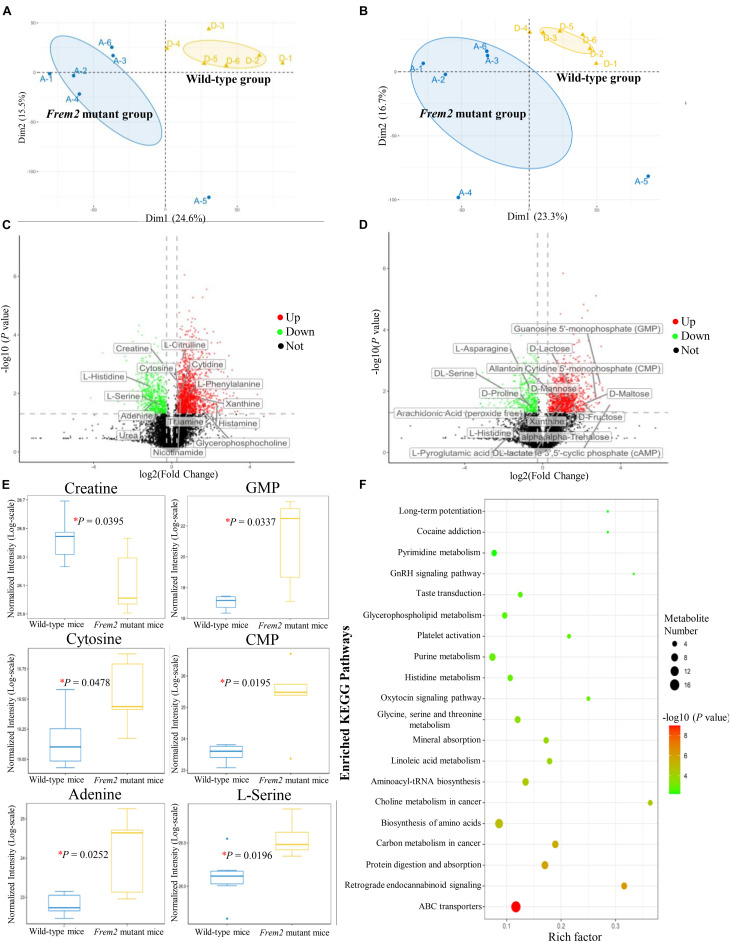
Metabolites profiles in fetal *Frem2^Δ*R725X/R2156W*^* mice. **(A,B)** Principal component analysis plots were performed under positive and negative modes. **(C,D)** Volcano plot showing the top 50 most different biologically significant metabolites under positive and negative modes. **(E)** Metabolites including creatine, guanosine 5′-monophosphate, cytosine, cytidine 5′-monophosphate, adenine, and L-serine were quantified in the embryos of *Frem2* mutant mice compared with wild-type mice by targeted LC-MS analyses (*P < 0.05). **(F)** KEGG metabolic pathway analysis of different metabolites.

**FIGURE 4:**
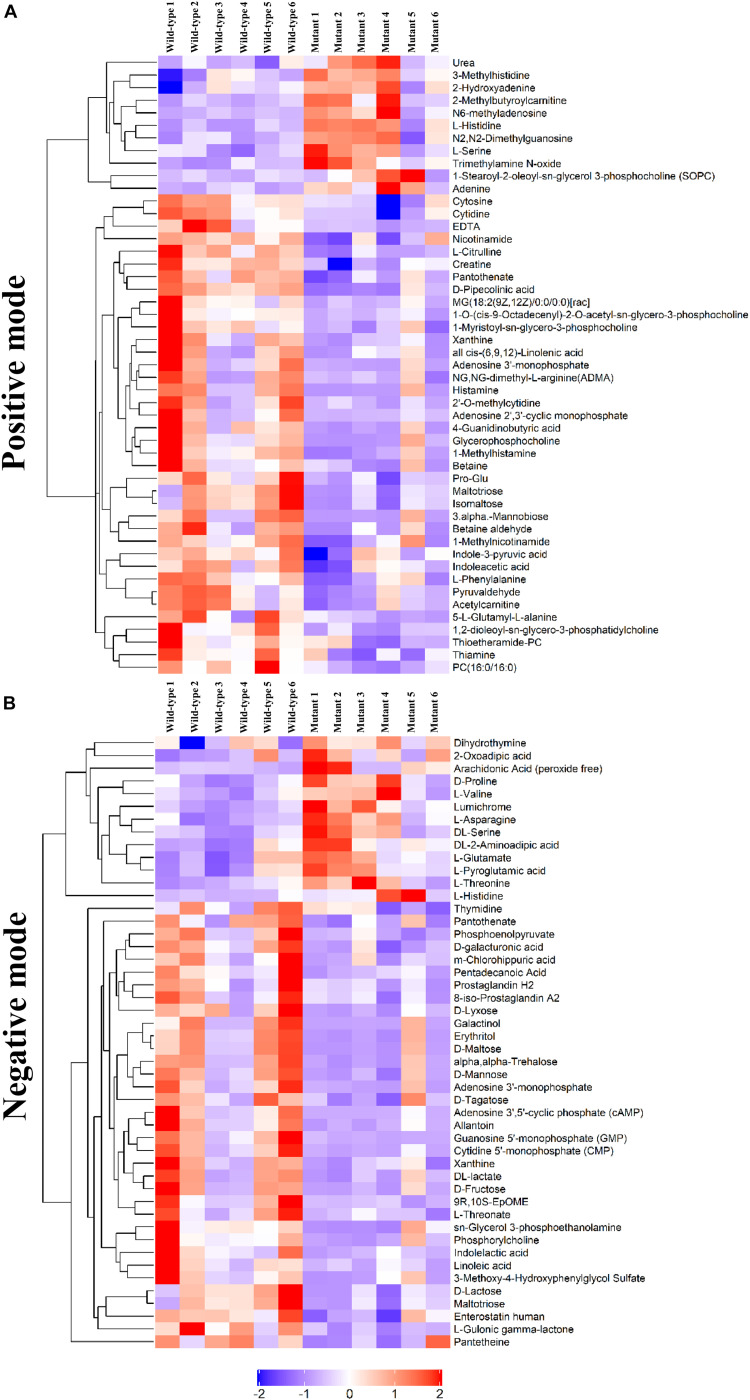
Heatmap of differentially expressed metabolites within fetal *Frem2^Δ*R725X/R2156W*^* mice under **(A)** positive and **(B)** negative modes.

To determine whether the metabolomic signatures were consistent with the transcriptomic signatures, a total of 92 metabolites that differed significantly between the mutant and wild-type embryos of E13.5 mice were identified by untargeted metabolomics analysis (Supplementary Table S2). Principal component analysis plots were performed for both positive and negative modes, showing that metabolite profiles in embryos from *Frem2* mutant mice with ocular hypoplasia were very different from those in embryos from wild-type mice (Figures 3A,B). We identified the top 50 most different biologically significant metabolites, displayed by volcano plots in Figures 3C,D. Heatmaps of differentially expressed metabolites within fetal *Frem2* mutant mice were visualized in Figure 4. Metabolites including creatine, guanosine 5′-monophosphate, cytosine, cytidine 5′-monophosphate, adenine, and L-serine were identified to be altered in the whole embryos of *Frem2* mutant mice by targeted LC-MS analyses (Figure 3E).

Metabolites determined in untargeted metabolomics analysis were screened and placed into the KEGG database to determine chemical and metabolic pathways that could be involved in the development of cryptophthalmos. A total of 153 possible pathways were found to be involved, covering 74 specific metabolites. KEGG metabolic pathway analysis of different metabolites annotated significant features related to ABC transporters and biosynthesis of amino acids, indicating that these substances could also be involved in the pathogenesis of cryptophthalmos (Table 1 and Figure 3F). Consistent with the KEGG analysis, real-time quantitative PCR confirmed the higher expression of ABC genes including *Abcc3*, *Abcc6*, and *Abcc9*, as well as genes contributing to the biosynthesis of amino acids in the embryos of *Frem2*^*R725X/R2156W*^ mice (Figure 2B).

**TABLE 1:** The significantly altered pathways including more than ≥5 different metabolites.

Map ID	Map name	Metabolites name
map02010	ABC transporters	L-Histidine, Cytidine, Maltotriose, L-Phenylalanine, L-Serine, Thiamine, Urea, Betaine, D-galacturonic acid, D-Lactose, D-Mannose, Erythritol, D-Maltose, L-Threonine, L-Valine, L-Glutamate
map01230	Biosynthesis of amino acids	L-Citrulline, L-Histidine, L-Phenylalanine, L-Serine, L-Asparagine, Phosphoenolpyruvate, DL-2-Aminoadipic acid, L-Threonine, L-Valine, L-Glutamate, 2-Oxoadipic acid
map04974	Protein digestion and absorption	L-Histidine, L-Phenylalanine, Histamine, L-Serine, L-Asparagine, L-Threonine, L-Valine, L-Glutamate
map00970	Aminoacyl-tRNA biosynthesis	L-Histidine, L-Phenylalanine, L-Serine, L-Asparagine, L-Threonine, L-Valine, L-Glutamate
map05230	Central carbon metabolism in cancer	L-Histidine, L-Phenylalanine, L-Serine, L-Asparagine, Phosphoenolpyruvate, L-Valine, L-Glutamate
map00230	Purine metabolism	Xanthine, Adenine, Adenosine 3′-monophosphate, Urea, Adenosine 2’,3′-cyclic monophosphate, Guanosine 5′-monophosphate (GMP), Adenosine 3′,5′-cyclic phosphate (cAMP)
map00260	Glycine, serine, and threonine metabolism	Creatine, Pyruvaldehyde, L-Serine, Betaine aldehyde, Betaine, L-Threonine
map04723	Retrograde endocannabinoid signaling	PC(16:0/16:0), 1-Stearoyl-2-oleoyl-sn-glycerol 3-phosphocholine (SOPC), cAMP, Arachidonic Acid, Prostaglandin H2, L-Glutamate
map04978	Mineral absorption	L-Phenylalanine, L-Serine, L-Asparagine, L-Threonine, L-Valine
map00591	Linoleic acid metabolism	PC(16:0/16:0), SOPC, all cis-(6,9,12)-Linolenic acid, Arachidonic Acid, Linoleic acid
map00330	Arginine and proline metabolism	Creatine, 4-Guanidinobutyric acid, Urea, D-Proline, L-Glutamate
map00590	Arachidonic acid metabolism	PC(16:0/16:0), SOPC, Arachidonic Acid, 8-iso-Prostaglandin A2, Prostaglandin H2
map00340	Histidine metabolism	L-Histidine, 1-Methylhistamine, 3-Methylhistidine, Histamine, L-Glutamate
map01210	2-Oxocarboxylic acid metabolism	L-Phenylalanine, DL-2-Aminoadipic acid, L-Valine, L-Glutamate, 2-Oxoadipic acid
map00240	Pyrimidine metabolism	Cytidine, Cytosine, Urea, Cytidine 5′-monophosphate (CMP), Thymidine
map00564	Glycerophospholipid metabolism	PC(16:0/16:0), Glycerophosphocholine, SOPC, sn-Glycerol 3-phosphoethanolamine, Phosphorylcholine

## Discussion

Ocular and eyelid development occurs in a step-wise fashion, and any misstep will be followed by the failure of subsequent steps (Tawfik et al., 2016). Our results confirmed that the phenotype of *Frem2* mutant embryonic mice shows consistent results with that of the adult mice characterized by abnormal eyelids, microphthalmia, and developmental defects in the anterior segment. In addition, these congenital ocular dysplasias begin to appear at E13.5.

This study has highlighted the impact of loss-of-function mutations of *Frem2* on the mouse embryonic metabolome. In particular, the data show connections between transcriptomic and metabolomic signatures in *Frem2* mutant mice, which is intriguing coincides with reductions in metabolites participating in amino acid metabolism and energy metabolism. Metabolites identified to be altered in the *Frem2* mutant mice by targeted LC-MS analyses, including guanosine 5′-monophosphate, cytosine, cytidine 5′-monophosphate, adenine, and L-serine, were all essential in the ocular development. Overall, we demonstrate that changes in metabolome composition may have contributed to the development of the cryptophthalmos phenotype in *Frem2* mutant mice.

As shown in the KEGG metabolic pathway analysis, 16 different metabolites have been revealed to be related to the map of ABC transporters, which constitute a ubiquitous protein superfamily forming the largest transporter gene family expressed in various body tissues (Lee et al., 2016). ABC transporters translocate a wide variety of substrates, hydrophobic compounds, and metabolites across extra and intracellular membranes and play critical roles in maintaining intracellular balance (Gottesman et al., 2002; Szakacs et al., 2006). Mutations in these genes contribute to disorders including cystic fibrosis, neurological disease, retinal degeneration, cholesterol and bile transport defects, anemia, and drug response (Dean et al., 2001). Mutations in the ABCC6 gene were proved to be responsible for connective tissue disorder (Saux et al., 2000) and suggested to play a role in the transportation of toxic metabolites to which connective tissue cells are sensitive (Fung et al., 2019). In addition to these activities, ABC transporters have been shown to mediate epithelial to mesenchymal transition, and the cystic fibrosis transmembrane conductance regulator has been proven to function as a chloride/anion channel in epithelial cells around the body (Meng et al., 2016). However, it is still unclear to what extent this transport effect is related to epithelial–mesenchymal coupling and how *FREM2* mutation affects ABC transporters.

Although connections between transcriptomic and metabolomic signatures have been shown in *Frem2* mutant mice, the results of our study should be cautiously interpreted within the context of C57BL/6J mice embryos, as postnatal validation in mice and humans should be further confirmed. Additional experiments are also required to study whether the metabolic alterations were paralleled to the genetic changes or served as upstream indicators in cryptophthalmos development. Meanwhile, experiments designed to clarify the chronology of the pathogenesis of cryptophthalmos are also essential for identifying when defects in the formation of the eye and other tissues occur.

Shifts in metabolome composition and functionality may have contributed to the cryptophthalmos phenotype. As a result, we should acknowledge the influence of both direct mutations in genes and indirect metabolic programming effects caused by their modulation in utero. This is suggestive of a cooperative action utilizing both metabolic detection and screening of pathogenic genes in prenatal diagnostic testing, as cryptothalmos is difficult to diagnose by prenatal B-ultrasound. Furthermore, as novel therapeutics of genome editing tools enter the market, it becomes increasingly important to consider their effects on the metabolome.

## Summary Statement

Metabolomic and transcriptomic signatures provide a map of metabolism in the *Frem2* mutant mouse model of cryptophthalmos. These data provide insights into further mechanistic studies of the pathogenesis of cryptophthalmos.

## Data Availability Statement

The data that support the findings of this study is available from the corresponding author upon reasonable request. The detected transcriptomics dataset and metabolomics datasets are available through Mendeley (https://data.mendeley.com/datasets/xfhsyssgrk/1).

## Ethics Statement

All mouse procedures were approved by the Institutional Animal Care and Use Committee of Zhongshan Ophthalmic Center, Sun Yat-sen University.

## Author Contributions

HL and XYZ designed the study. XYZ, RW, XLZ, DW, and MD performed the experiments. XYZ analyzed the data and wrote the manuscript. TW, XW, DL, JW, and WL critically revised the manuscript. All authors discussed the results and commented on the manuscript.

## Conflict of Interest

The authors declare that the research was conducted in the absence of any commercial or financial relationships that could be construed as a potential conflict of interest.

## Footnotes

1 http://david.abcc.ncifcrf.gov/

2 www.metaboanalyst.ca

3 http://www.genome.jp/kegg/

## Supplementary Material

The detected transcriptomics dataset and metabolomics datasets are available through Mendeley (https://data.mendeley.com/datasets/xfhsyssgrk/1).

## Abbreviations

ABC transporters: ATP binding cassette transporters
ESI: electrospray ionization
H&E: hematoxylin-eosin
KEGG: Kyoto Encyclopedia of Genes and Genomes
LC-MS: liquid chromatography-mass spectrometry
MRM: multiple reaction monitoring
MS: mass spectrometry
PCR: polymerase chain reaction
RNA-seq: RNA sequencing
TOF: time-of-flight
UHPLC: ultra high-performance liquid chromatography
VIP: variable influence on projection.
