# Supplementary material for: The Metabolic Reprogramming of Frem2 Mutant Mice Embryos in Cryptophthalmos Development
Source: Front Cell Dev Biol. 2021 Jan 8;8:625492. doi: 10.3389/fcell.2020.625492 (PMC7820765; doi:10.3389/fcell.2020.625492)
Supplement: Supplementary file 1 [file Data_Sheet_1.docx]

**Table S1. The primer sequences used to validate expression changes of selected genes by the real-time quantitative PCR technique.**

|  | **Genes** | **Primers name** | **Sequence (5’-3’)** |
| --- | --- | --- | --- |
| 1 | *Stat5a*  ENSMUSG00000004043 | Stat5a-F | CGCCAGATGCAAGTGTTGTAT |
|  |  | Stat5a-R | TCCTGGGGATTATCCAAGTCAAT |
| 2 | *Adssl1*  ENSMUSG00000011148 | Adssl1-F | CTCACCTTGTGTTCGACTTCC |
|  |  | Adssl1-R | AGCAAAGCCCTTGAGCCTTTT |
| 3 | *Dpys*  ENSMUSG00000022304 | Dpys-F | CCACAGGGACGACTTCTCATC |
|  |  | Dpys-R | CGCTGCATCTAGGATTCGCA |
| 4 | *Cbs*  ENSMUSG00000024039 | Cbs-F | CCAGGCACCTGTGGTCAAC |
|  |  | Cbs-R | GGTCTCGTGATTGGATCTGCT |
| 5 | *Ldhb*  ENSMUSG00000030246 | Ldhb-F | CATTGCGTCCGTTGCAGATG |
|  |  | Ldhb-R | GGAGGAACAAGCTCCCGTG |
| 6 | *Pm20d1*  ENSMUSG00000042251 | Pm20d1-F | CTTCTCTTTTTCGCTACGGTCT |
|  |  | Pm20d1-R | CACCTTTCAGCGCCTCTTTTAT |
| 7 | *Chchd10*  ENSMUSG00000049422 | Chchd10-F | CAGCCGGGTCTTATGGCTC |
|  |  | Chchd10-R | CAGGCTCTGAATTTCCCCCAC |
| 8 | *Hk1*  ENSMUSG00000037012 | Hk1-F | CGGAATGGGGAGCCTTTGG |
|  |  | Hk1-R | GCCTTCCTTATCCGTTTCAATGG |
| 9 | *Abcc3*  ENSMUSG00000020865 | Abcc3-F | CTGGGTCCCCTGCATCTAC |
|  |  | Abcc3-R | GCCGTCTTGAGCCTGGATAAC |
| 10 | *Abcc9*  ENSMUSG00000030249 | Abcc9-F | CACACCGGAGTGCAATCAAAA |
|  |  | Abcc9-R | ATCCATTTGTCACCTTTATGGCA |
| 11 | *Abcc6*  ENSMUSG00000030834 | Abcc6-F | TGCGGCCTATCACTTGCTC |
|  |  | Abcc6-R | CCAGCACCATTTTGGTTTTGAA |

**Table S2. Identified differential metabolites between *Frem2* mutant embryonic mice and wild-type embryonic mice.**

| Metabolites | VIP | P-value |
| --- | --- | --- |
| D-Pipecolinic acid | 2.0914 | 0.0002 |
| L-Citrulline | 1.2910 | 0.0007 |
| Creatine | 5.7767 | 0.0033 |
| Pantothenate | 8.2828 | 0.0036 |
| L-Gulonic gamma-lactone | 2.0087 | 0.0042 |
| Guanosine 5'-monophosphate (GMP) | 1.4002 | 0.0054 |
| Isomaltose | 3.3259 | 0.0054 |
| L-Histidine | 5.4421 | 0.0055 |
| D-galacturonic acid | 2.5767 | 0.0059 |
| 1-Methylhistamine | 4.0986 | 0.0060 |
| Pro-Glu | 1.4814 | 0.0062 |
| Cytidine | 4.9596 | 0.0079 |
| Maltotriose | 5.4335 | 0.0079 |
| PC(16:0/16:0) | 7.1187 | 0.0082 |
| Trimethylamine N-oxide | 1.8103 | 0.0090 |
| L-Asparagine | 2.1191 | 0.0090 |
| Lumichrome | 1.8206 | 0.0094 |
| D-Lactose | 2.3461 | 0.0100 |
| L-Phenylalanine | 1.3675 | 0.0100 |
| 1,2-dioleoyl-sn-glycero-3-phosphatidylcholine | 11.7941 | 0.0100 |
| D-Lyxose | 1.2620 | 0.0106 |
| Acetylcarnitine | 8.9099 | 0.0123 |
| Cytosine | 5.0319 | 0.0127 |
| 4-Guanidinobutyric acid | 2.1654 | 0.0130 |
| Pyruvaldehyde | 1.0910 | 0.0132 |
| 3-Methylhistidine | 1.3494 | 0.0133 |
| Cytidine 5'-monophosphate (CMP) | 3.0063 | 0.0142 |
| Nicotinamide | 17.7999 | 0.0145 |
| EDTA | 1.3976 | 0.0146 |
| Xanthine | 1.2158 | 0.0148 |
| Enterostatin human | 1.6815 | 0.0160 |
| Phosphoenolpyruvate | 1.8992 | 0.0164 |
| D-Mannose | 7.7956 | 0.0194 |
| Allantoin | 2.3443 | 0.0196 |
| N6-methyladenosine | 5.2980 | 0.0200 |
| Adenine | 1.3617 | 0.0219 |
| 2-Methylbutyroylcarnitine | 9.0893 | 0.0222 |
| Erythritol | 1.5295 | 0.0228 |
| 5-L-Glutamyl-L-alanine | 1.4469 | 0.0228 |
| Indoleacetic acid | 1.7848 | 0.0232 |
| Histamine | 6.6504 | 0.0236 |
| L-Serine | 1.1246 | 0.0239 |
| 9R,10S-EpOME | 1.1938 | 0.0240 |
| alpha,alpha-Trehalose | 1.1664 | 0.0247 |
| D-Maltose | 4.1148 | 0.0254 |
| 1-Myristoyl-sn-glycero-3-phosphocholine | 2.5027 | 0.0255 |
| N2,N2-Dimethylguanosine | 1.8428 | 0.0263 |
| D-Fructose | 1.0783 | 0.0267 |
| DL-Serine | 1.9521 | 0.0276 |
| Adenosine 3',5'-cyclic phosphate (cAMP) | 1.2533 | 0.0287 |
| D-Proline | 5.0911 | 0.0297 |
| DL-2-Aminoadipic acid | 1.3905 | 0.0301 |
| 1-Methylnicotinamide | 4.1565 | 0.0311 |
| Betaine aldehyde | 3.7964 | 0.0314 |
| Adenosine 3'-monophosphate | 2.6778 | 0.0315 |
| Thiamine | 2.1447 | 0.0324 |
| 2'-O-methylcytidine | 1.5505 | 0.0325 |
| Pentadecanoic Acid | 3.2724 | 0.0338 |
| 3.alpha.-Mannobiose | 7.7892 | 0.0341 |
| Glycerophosphocholine | 8.4536 | 0.0364 |
| Urea | 1.3275 | 0.0375 |
| Arachidonic Acid (peroxide free) | 1.6287 | 0.0394 |
| L-Threonate | 1.1437 | 0.0397 |
| DL-lactate | 1.6829 | 0.0402 |
| Indolelactic acid | 1.7548 | 0.0410 |
| Indole-3-pyruvic acid | 1.0414 | 0.0425 |
| 1-O-(cis-9-Octadecenyl)-2-O-acetyl-sn-glycero-3-phosphocholine | 1.6029 | 0.0433 |
| L-Pyroglutamic acid | 3.1723 | 0.0433 |
| Adenosine 2',3'-cyclic monophosphate | 1.0695 | 0.0478 |
| 2-Hydroxyadenine | 6.1714 | 0.0503 |
| MG(18:2(9Z,12Z)/0:0/0:0)[rac] | 2.3141 | 0.0507 |
| L-Threonine | 3.7829 | 0.0521 |
| Thioetheramide-PC | 11.5818 | 0.0531 |
| L-Valine | 4.7591 | 0.0536 |
| Betaine | 8.1964 | 0.0569 |
| sn-Glycerol 3-phosphoethanolamine | 2.0723 | 0.0586 |
| Galactinol | 4.9325 | 0.0612 |
| 8-iso-Prostaglandin A2 | 1.0203 | 0.0623 |
| Phosphorylcholine | 1.2098 | 0.0626 |
| m-Chlorohippuric acid | 1.1783 | 0.0651 |
| Linoleic acid | 28.9083 | 0.0656 |
| NG,NG-dimethyl-L-arginine(ADMA) | 9.0487 | 0.0681 |
| Prostaglandin H2 | 1.7094 | 0.0725 |
| D-Tagatose | 1.8392 | 0.0763 |
| 1-Stearoyl-2-oleoyl-sn-glycerol 3-phosphocholine (SOPC) | 5.1027 | 0.0807 |
| Dihydrothymine | 2.0652 | 0.0821 |
| L-Glutamate | 5.6643 | 0.0849 |
| 3-Methoxy-4-Hydroxyphenylglycol Sulfate | 1.4083 | 0.0917 |
| all cis-(6,9,12)-Linolenic acid | 1.0519 | 0.0941 |
| Thymidine | 5.3391 | 0.0949 |
| 2-Oxoadipic acid | 2.2318 | 0.0964 |
| Pantetheine | 1.6436 | 0.0997 |

VIP, variable importance in the projection.
